# A Case of Thrombocytopenia due to Odontogenic Infection

**DOI:** 10.5681/joddd.2011.033

**Published:** 2011-12-19

**Authors:** Dinesh Kumar Verma, Ritesh Rajan

**Affiliations:** ^1^Reader, Department of Oral and Maxillofacial Surgery, Sharad Pawar Dental College and Hospital, Sawangi, Wardha, Maharashtra, India

**Keywords:** Thrombocytopenia, odontogenic infections, sepsis

## Abstract

Thrombocytopenia in surgical patients is a potentially serious condition,faced by surgeons. A close relationship between sepsis and thrombocytopenia has been suggested. Thrombocytopenia has even been suggested to be indicative of an acute infection. Platelet count in a septicemic patient may also serve as a prognostic tool. There are many reports of thrombocy-topenia due to septicemia in the literature but the occurrence of thrombocytopenia in maxillofacial infections is rare. Thrombocytopenia in a patient with odontogenic infection presents unique diagnostic and management challenges. A case report of an adult male patient with odontogenic infection, who developed life-threatening thrombocytopenia, is presented.

## Introduction

Odontogenic infections can cause life-threatening complications, such as cavernous sinus thrombosis, mediastinitis, aspiration pneumonia and

septicemia. Coagulation abnormalities, ranging from thrombocytopenia to a fulminant state of disseminated intravascular coagulation (DIC), are well recognized in ICU patients with septicemia. The incidence of thrombocytopenia in ICU patients ranges from 15% to 58%, depending on the type of population and the threshold used to define thrombocytopenia.^1^It has been suggested that thrombocytopenia reflects the severity and progression of the underlying infection, and may serve as an indicator of acute infection. ^2 - 4^ It can also serve as a prognostic indicator.^5 - 7^ Although thrombocytopenia, in the critically ill, is not a disease process per se, it may increase mortality in several ways. It can result in a mild, moderate, or severe hemorrhagic disorder, which could enhance the risk of morbidity and mortality in critically ill surgical patients.

## Case report

A middle-aged male patient reported to the Emergency Department of Sharad Pawar Dental College and Hospital, Wardha, with a complaint of pain and swelling over left side of the face, left upper neck, pyrexia and trismus. The patient gave a history of continuous, low-grade throbbing pain in relation to the left mandibular first molar of a two-month duration with exacerbation of symptoms over the week prior to presentation. The episode was immediately followed by swelling over the left cheek, pyrexia and trismus, which rapidly progressed to involve the left infraorbital and submandibular regions. The patient had been successfully treated for pulmonary tuberculosis 20 years back with antituberculosis drugs. Apart from that, the patient did not have any other significant dental, medical, drug or family history.

On examination, the swelling was tender, warm, tense and extended over the left cheek, left infraorbital and left submandibular regions. There were no draining sinuses. The patient also had trismus (interincisal mouth opening of 10 mm). The mandibular left first molar was carious and tender. The buccal vestibule was obliterated in relation to the molar. General examination revealed a temperature of 38.6°C, a heart rate of 96/min, respiratory rate of 24/min, and a BP of 108/80 mmHg. The aspirate from the left buccal space was purulent.

Routine hematological investigations and cell count studies revealed the following: Total leucocyte count, 15600/cu mm; platelet count, 68000/µL, and Hb, 9.0 gr. The platelet count was repeated to exclude the possibility of pseudothrombocytopenia. Prothrombin time was within normal limits. Purulent aspirate and blood samples were sent for microbiological examination. The condition was diagnosed as left buccal, submandibular and canine space infection, with sepsis and thrombocytopenia.

Hematology consultation was sought for joint management of the condition. Amoxicillin with clavulanic acid, amikacin and metronidazole were started along with crystalloids to correct dehydration. Incision and drainage procedure was carried out through submandibular and vestibular incisions. The mandibular left first molar was extracted.

Five hours into the post-operative period, the patient complained of difficulty in breathing. Mild oozing of blood from the intraoral and extraoral wounds was observed. PA chest radiography was not suggestive of acute respiratory distress syndrome. PA and lateral neck radiographs did not show features of parapharyngeal and retropharyngeal extension of infection. Pulse oximetry revealed normal saturation. Considering the possibility of laryngeal edema and aspiration of blood, tracheotomy was performed and cuffed tracheotomy tube was inserted.About 8 hours post-operatively, a drop in blood pressure to 84/54 mmHg was observed, which could only be raised and maintained with dopamine infusion.On the second post-operative day, total leucocyte count (19000/mm^3^) and platelet count (28000/µL) showed significant deviation from the baseline values.The purulent aspirate revealed gram-positive cocci; however, blood culture was negative for microorganisms. Eight units of platelet concentrate and 1 unit of whole blood were transfused. On the third day, the platelet count rose to 156000/µL and the total leucocyte count showed a drop to 13000/mm^3^.The patient was afebrile, able to maintain blood pressure within the normal range without dopamine infusion and showed a decrease in the extraoral swelling.

The patient continued to improve till the seventh day, when the platelet count again dropped to 48000/µL; total leucocyte count was 10,000/mm^3^. PT and aPTT were found to be within acceptable limits. Although there was no active bleeding from any of the wounds, 4 units of platelet concentrate and 1 unit of whole blood were transfused to reduce the risk of fresh bleeding. On the eighth day, the platelet count showed a further drop to 38000/µL; however, the total leucocyte count was 10000/mm^3^. Four extra units of platelet concentrate and 1 unit of whole blood were transfused. Despite falling platelet counts, patient’s general condition and the wound showed consistent improvement. The development of alloantibodies to platelets was suspected, for which, prednisolone 100 mg IV b.d. was instituted, which was administered for 5 days. Further platelet transfusions were withheld.

From the tenth day on, platelet count showed a steady improvement. The patient was discharged on the fifteenth day with platelet count of 174000/µL, total leucocyte count of 8000/mm^3^ and healed wounds. Four months later, the patient reported to the Department of Oral and Maxillofacial Surgery for extraction of right maxillary first molar. Basic hematological and cell count screening did not reveal any abnormalities. Extraction was carried out and the post-operative period was uneventful.

## Discussion

Septicemia is a potentially lethal condition associated with a mortality rate of 52–60% in severe sepsis and 55–66% in culture-negative severe sepsis.^8^

The characteristics of different stages of sepsis are:^9^

Stage I—Systemic Inflammatory Response Syndrome (SIRS). Two or more of the following:

1. temperature more than 38°C or less than 36°C
2. heart rate more than 90/min
3. respiratory rate more than 20/min
4. white blood count more than 12000/mm^3^ or less than 4000/mm^3^ or presence of more than 10% of band cells

Stage II—Sepsis. SIRS with a culture-documented infection

Stage III—Severe sepsis. Sepsis with organ dysfunction, hypotension, or hypoperfusion (lactic acidosis, oliguria, hypoxemia or acute alteration in mental status)

Stage IV—Septic shock. Hypotension (despite fluid resuscitation) with evidence of hypoperfusion.

Sepsis can be caused by any microorganism. However, patients with clinically suspected sepsis, but without positive culture documentation, are at equally high risk of death. Early diagnosis and aggressive treatment is important because patients with sepsis may also develop cardiopulmonary, renal, coagulative or neurologic complications.

A close relationship between sepsis and thrombocytopenia has been suggested. Sepsis was found to be one of the predominant risk factors for the development of thrombocytopenia.^4^ Thrombocytopenia may reflect the severity and progression of an underlying pathologic condition. It may even serve as a prognostic indicator in severe infections.^5 - 7^

Occurrence of thrombocytopenia in sepsis has been thought to be due to increased platelet destruction,^10^ impairment of platelet production,^11^ or adherence of platelets to damaged endothelium.^12^ Consumptive coagulopathy like DIC can also lead to a decrease in platelet count.^13^ Immune mechanisms have also been suggested, which lead to thrombocytopenia in a patient with sepsis.^13^ Antibiotics like vancomycin and piperacillin, used frequently in severe infections, can also cause a decrease in platelet counts.^14^

The peripheral blood count is the key to establish the presence and severity of thrombocytopenia. The screening tests of hemostasis will be normal unless thrombocytopenia is associated with conditions affecting hemostasis (e.g. liver disease and DIC). Laboratory tests indicating DIC^15^ are prolonged aPTT and PT, decreased platelet counts and fibrinogen concentration, elevated D-dimer and fibrin split products. Bone marrow aspiration may be indicated if abnormalities other than thrombocytopenia are noted on the peripheral smear. Routine bone marrow examination in septic patients may not be helpful for diagnosis of thrombocytopenia as it may be caused by platelet sequestration or destruction.^4^ In vitro platelet clumping in the presence of EDTA and platelet satellitism around neutrophils may lead to pseudothrombocytopenia.^16^

The management of an odontogenic infection complicated by thrombocytopenia presents a tricky situation to the surgeon. Maxillofacial space infections usually require incision and drainage. However, the surgeon may be reluctant to operate due to presence of thrombocytopenia. Sepsis, on the other hand, will probably continue unless surgical drainage is established. The risk of spontaneous bleeding does not increase until the platelet count falls below 20000/µL.^17^ Platelet transfusions are frequently employed to increase the platelet count in critically ill patients. However, patients with sepsis respond suboptimally to platelet transfusions due to continued platelet destruction, DIC, hypothermia or ‘stunned’ transfused platelets.^4^ Laboratory findings alone should not be the criteria for platelet transfusion. Patients who are actively bleeding, who require an invasive procedure, or who are at risk of bleeding complications (post-operative patients) should undergo platelet transfusion.^18^ In a hemorrhagic patient, the platelet count should be kept in the range of 50000-70000/µL.^19^ Discretion should be exercised in administering multiple platelet transfusions because of the development of alloantibodies.^20^ Finally, the number of platelet units to be transfused should be determined in a case-specific manner.

## Conclusion

Thrombocytopenia increases the risk of bleeding, alters the plan for care and may serve as an indicator of severity of infection. In patients with severe infection, the platelet count may help monitor the progression of disease.
